# Neonatal Exposure to Lipopolysaccharide Promotes Neurogenesis of Subventricular Zone Progenitors in the Developing Neocortex of Ferrets

**DOI:** 10.3390/ijms241914962

**Published:** 2023-10-06

**Authors:** Kazuhiko Sawada, Shiori Kamiya, Tetsuya Kobayashi

**Affiliations:** 1 Department of Nutrition, Faculty of Medical and Health Sciences, Tsukuba International University, Tsuchiura 300-0051, Japan; 2 Department of Regulation Biology, Faculty of Science, Saitama University, Saitama 338-8570, Japan; sk.dietitian2017@gmail.com (S.K.); tkoba@mail.saitama-u.ac.jp (T.K.)

**Keywords:** toll-like receptor, intermediate progenitors, Tbr2, Ctip2, carnivores

## Abstract

Lipopolysaccharide (LPS) is a natural agonist of toll-like receptor 4 that serves a role in innate immunity. The current study evaluated the LPS-mediated regulation of neurogenesis in the subventricular zone (SVZ) progenitors, that is, the basal radial glia and intermediate progenitors (IPs), in ferrets. Ferret pups were subcutaneously injected with LPS (500 μg/g of body weight) on postnatal days (PDs) 6 and 7. Furthermore, 5-ethynyl-2′-deoxyuridine (EdU) and 5-bromo-2′-deoxyuridine (BrdU) were administered on PDs 5 and 7, respectively, to label the post-proliferative and proliferating cells in the inner SVZ (iSVZ) and outer SVZ (oSVZ). A significantly higher density of BrdU single-labeled proliferating cells was observed in the iSVZ of LPS-exposed ferrets than in controls but not in post-proliferative EdU single-labeled and EdU/BrdU double-labeled self-renewing cells. BrdU single-labeled cells exhibited a lower proportion of Tbr2 immunostaining in LPS-exposed ferrets (22.2%) than in controls (42.6%) and a higher proportion of Ctip2 immunostaining in LPS-exposed ferrets (22.2%) than in controls (8.6%). The present findings revealed that LPS modified the neurogenesis of SVZ progenitors. Neonatal LPS exposure facilitates the proliferation of SVZ progenitors, followed by the differentiation of Tbr2-expressing IPs into Ctip2-expressing immature neurons.

## 1. Introduction

Epidemiological and preclinical studies have shown that maternal immune activation (MIA) by infections with bacteria and viruses such as SARS-CoV-2 increases the risk of neurodevelopmental disorders, including autism spectrum disorder (ASD), schizophrenia, attention-deficit/hyperactivity disorder, and cognitive dysfunction [1,2,3]. Investigating structural and functional brain abnormalities in animal models of MIA could be used to understand the pathogenesis of various neurodevelopmental disorders. Lipopolysaccharide (LPS), an endotoxin localized in the outer membrane of Gram-negative bacteria, is a natural agonist of toll-like receptor 4 (TLR4) and serves a role in initiating innate immune responses [4,5]. LPS stimulates microglia to release proinflammatory cytokines, tumor necrosis factor-α (TNF-α) and interleukin 6 (IL-6), and the anti-inflammatory, cytokine interleukin 10, through the activation of TLR4 signaling in the central nervous system (CNS) [4,6]. LPS has been used to produce animal models of MIA as well as polyinosinic:polycytidylic acid (Poly I:C) [7]. Prenatal exposure to LPS has been shown to induce histoarchitectural alterations in the mature cerebral cortex, such as overall hypomyelination and lesser parvalbumin-expressing neurons in the prefrontal cortex and abnormal organization of minicolumns in the anterior cingulate and somatosensory cortices in rodent offspring with ASD and/or schizophrenia-like social behavior impairments [8,9,10,11].

Ferrets are gyrencephalic animals with a higher proportion of white matter to gray matter, similar to humans [12], but they are distinct in their experience of cortical sulcogyrogenesis during the first three postnatal weeks [13,14]. Gyrification abnormalities have been extensively documented in human patients with neurodevelopmental disorders such as ASD and schizophrenia [15,16,17]. In ferrets, valproic acid (VPA) exposure during cortical neurogenesis causes ASD-like social behavior impairments [18], similar to that observed in rodents [19], and abnormal sulcal infolding in multimodal-association cortical regions by modification of the cortical thickness of the sulcal bottoms [20]. Such gyrification abnormalities are involved in VPA-induced proliferation of subventricular zone (SVZ) progenitors, followed by their differentiation into cortical neurons [21]. Although LPS exposure, through TLR4 activation, modulates adult hippocampal neurogenesis and neurogenesis for brain repair following injury [22,23,24,25,26,27], the influence of LPS on SVZ progenitor neurogenesis in relation to the histoarchitectures of the cerebral cortex is poorly understood. Therefore, the present study evaluated LPS-mediated regulation of the proliferation, maintenance, and/or differentiation of SVZ progenitors, that is, the basal radial glia (bRG) and intermediate progenitors (IPs). Ferrets have an advantageous characteristic in that a self-renewable neural stem cell, bRG (also known as outer radial glia), emerges abundantly in the inner SVZ (iSVZ) and outer SVZ (oSVZ) of the developing cerebral cortex, similar to other gyrencephalic mammals, such as humans and non-human primates, but unlike lissencephalic rodents [28,29]. bRG, which contributes to the vast expansion of the cerebral cortex in relation to sulcogyrogenesis [30], emerges abundantly at mid-gestation in humans and non-human primates, as well as during the initial postnatal weeks of age in ferrets [28,29]. Therefore, in the current investigation, ferret infants were exposed to LPS on postnatal days (PDs) 6 and 7, which correspond to the neurodevelopmental stage of the cerebral cortex in primates at mid-gestation. Therefore, this study’s findings can be extrapolated to humans as an MIA model. Using a ferret model would further allow the investigation of gyrification abnormalities caused by neonatal exposure to LPS as the next step.

## 2. Results

### 2.1. Gross Structures of the Brain

**Figure 1:**
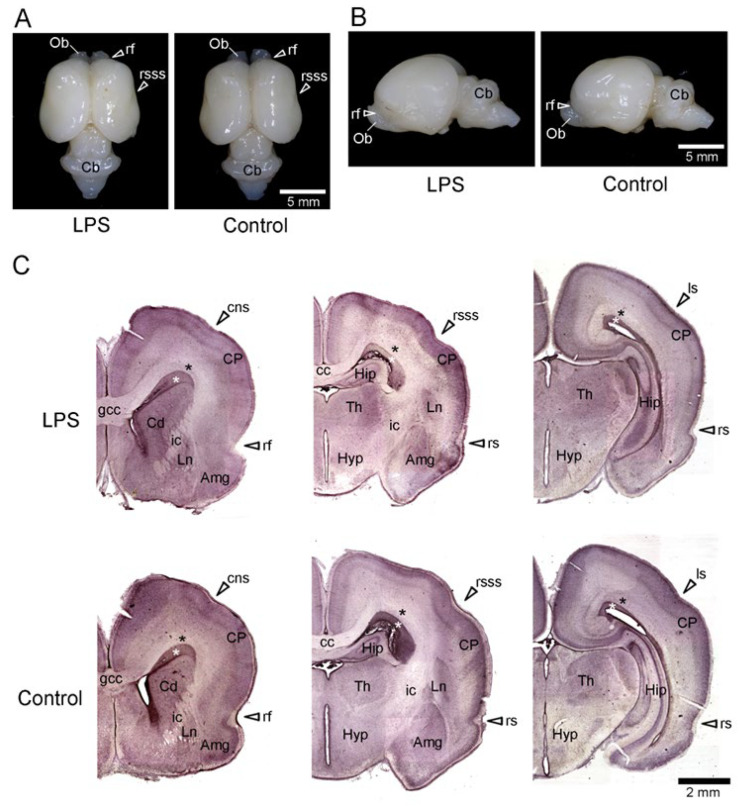
Gross images of the brains of LPS-exposed and control ferrets on postnatal day 7. (**A**) Dorsal view of the brain. (**B**) Lateral view of the left side of the brain. (**C**) Coronal hematoxylin-stained sections are shown from left to right in the rostrocaudal order. White asterisk indicates the inner subventricular zone (iSVZ). Black asterisk indicates the outer subventricular zone (oSVZ). Amg, amygdaloid complex; cc, corpus callosum; Cb, cerebellum; Cd, caudate nucleus; cns, coronal sulcus; CP, cortical plate; gcc, genu of corpus callosum; Hip, hippocampus; Hyp, Hypothalamus; ic, internal capsule; Ln, lentiform nucleus; ls, lateral sulcus; rf, rhinal fissure; rs, rhinal sulcus; rsss, rostral suprasylvian sulcus; Th, thalamus.

Gross anatomical images of the brains of LPS-exposed and control ferrets on postnatal day (PD) 7 are shown in Figure 1A,B. The rostral suprasylvian sulcus (rsss) was identified as a narrow indentation on the dorsal surface of the cerebral cortex (Figure 1A). The rhinal fissure (rf) was infolded to demarcate the olfactory bulb and prefrontal cortical regions (Figure 1A,B). The coronal sulcus (ss), rhinal sulcus (rs), and lateral sulcus (ls) were observed on the coronal images of the hematoxylin-stained histological sections (Figure 1C). There was no difference in sulcal infolding between the LPS-exposed and control ferrets (Figure 1A–C). Moreover, when comparing brain weights, the results of the Student’s *t*-test did not reveal any difference between the LPS-exposed (986 ± 63 mg, *n* = 3) and control (1114 ± 72 mg, *n* = 3) groups. Thus, the gross structure of the brain was not altered immediately after LPS exposure in ferret neonates.

### 2.2. Densities of EdU+, BrdU+, and EdU+/BrdU+ Cells

**Figure 2:**
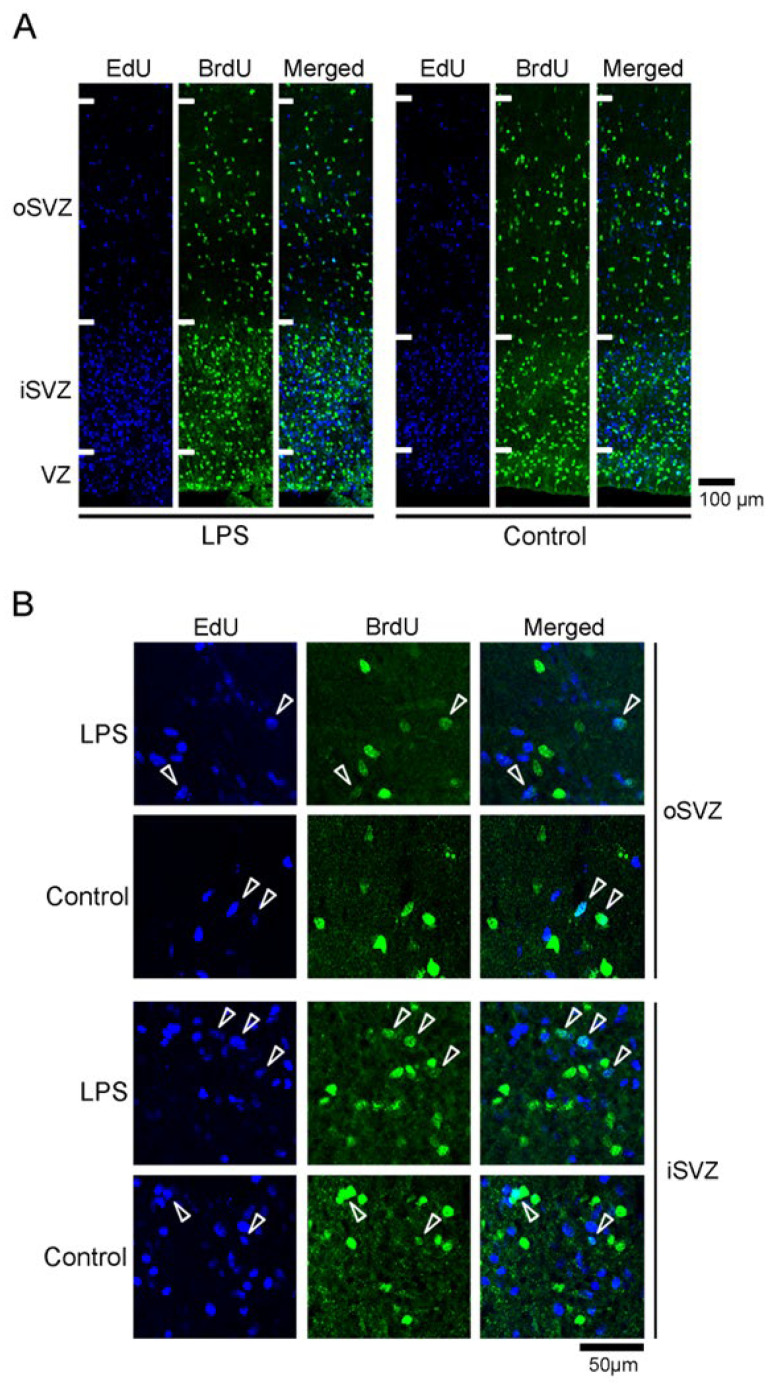
EdU and BrdU labeling in the premature cortex in the LPS-exposed and control ferrets on postnatal day 7. (**A**) Low-magnification images of the outer subventricular zone (oSVZ) and inner subventricular zone (iSVZ). Positions for capturing immunofluorescent images are shown in Figure S1A. (**B**) High-magnification images of the oSVZ and iSVZ. Positions for capturing immunofluorescent images are shown in Figure S1B. Open arrowheads indicate EdU/BrdU double-labeled cells. VZ, ventricular zone.

**Figure 3:**
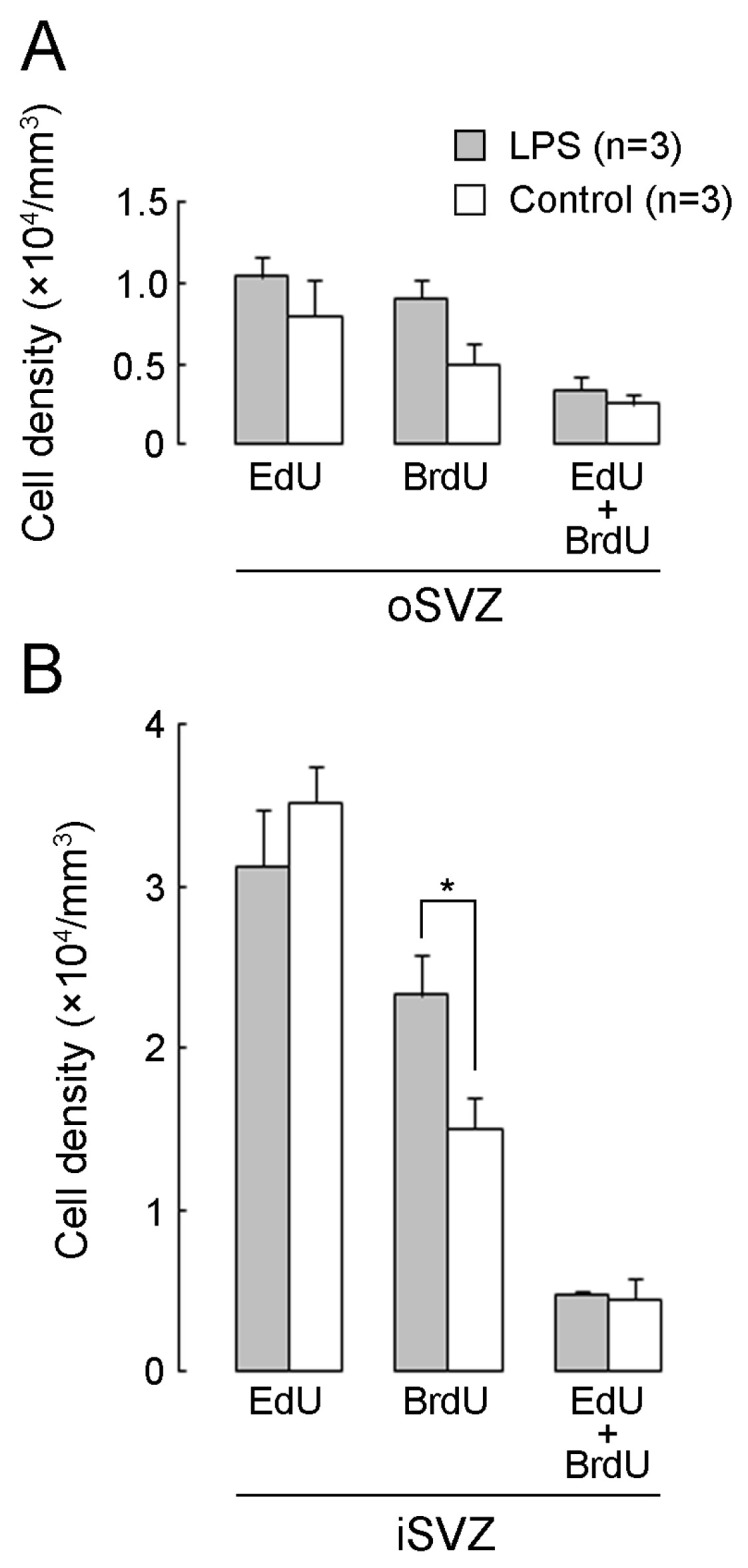
Densities of EdU single-, BrdU single-, and EdU/BrdU double-labeled cells in the premature cortex of the LPS-exposed and control ferrets on postnatal day 7. (**A**) Outer subventricular zone (oSVZ). (**B**) Inner subventricular zone (iSVZ). Data are shown as means ± standard deviations. Significance is indicated using Scheffe’s test at * *p* < 0.001; number of ferrets = 3 per group.

In this paper, we use the terms “immunopositive” or “positive labeling” (+) to refer to cells labeled with marker antigens or thymidine analogs at high and medium levels and “immunonegative” or “no labeling” (−) for cells labeled at low levels or unlabeled cells. 5-ethynyl-2′-deoxyuridine (EdU) labeling was observed in cells that proliferated on PD 5 24 h before the first injection of LPS. EdU+ cells were abundant in the iSVZ and diffused throughout the oSVZ of both the LPS-exposed and control ferrets (Figure 2A). Cells with 5-bromo-2′-deoxyuridine (BrdU) labeling, which proliferated on PD 7 immediately following the second injection of LPS, were observed diffusely throughout the iSVZ to the oSVZ in both groups (Figure 2A). Although the majority of these cells were single-labeled with EdU or BrdU, a small proportion of cells was EdU/BrdU double-labeled (EdU+/BrdU+) (Figure 2B). EdU+/BrdU+ cells experienced two rounds of cell division at a 48 h interval, leading to labeling of the self-renewing bRG [31]. The densities of EdU+ or EdU+/BrdU+ cells in the iSVZ and oSVZ were not statistically different between the LPS-exposed and control ferrets (Figure 3A,B), suggesting that LPS did not influence the self-renewal of the bRG. In contrast, BrdU+ cells were significantly denser in the iSVZ of LPS-exposed ferrets than in control ferrets, according to results of Scheffe’s test (*p* < 0.001), following significant effects of LPS exposure (*F_[1,4]_* = 22.573, *p* < 0.01) and SVZ regions (*F_[1,4]_* = 98.734, *p* < 0.001) by two-way repeated-measures ANOVA (Figure 3A). Thus, LPS promoted the proliferation of SVZ progenitors in the iSVZ.

### 2.3. Proportion of Immunostaining for Various Markers in Thymidine Analog-Labeled Cells

EdU and BrdU labeling was performed with immunofluorescence staining for various marker antigens, such as cleaved caspase 3 (cCasp3), proliferating cell nuclear antigen (PCNA), Ki67, phosphohistone H3 (PH3), T-box brain protein 2 (Tbr2), paired box 6 (Pax6), Olig2, Cux1, and Ctip2.

#### 2.3.1. Proportion of cCasp3 Immunostaining

**Figure 4:**
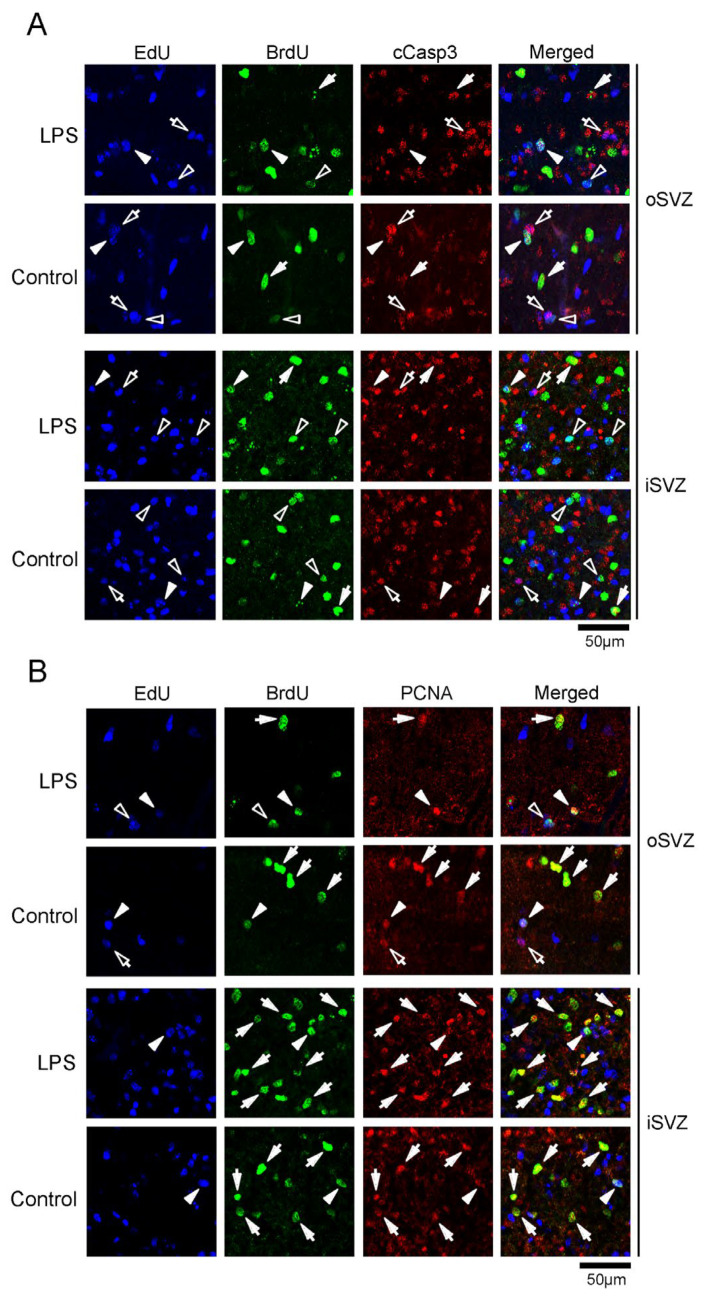
Immunofluorescence for cleaved caspase 3 (cCasp3) and proliferating cell nuclear antigen (PCNA) with EdU and BrdU labeling in the premature cortex of the LPS-exposed and control ferrets on postnatal day 7. Positions for capturing immunofluorescent images are shown in Figure S1B. (**A**) cCasp3 immunofluorescence with EdU and BrdU labeling in the outer and inner subventricular zones (oSVZ and iSVZ). Open arrowheads, EdU/BrdU double-labeled cells; closed arrowheads, cCasp3-immunopositive cells with EdU/BrdU double-labeling; open arrows, cCasp3-immunopositive cells with EdU single-labeling; closed arrows, cCasp3-immunopositive cells with BrdU single-labeling. (**B**) PCNA immunofluorescence with EdU and BrdU labeling in the oSVZ and iSVZ. Open arrowheads, EdU/BrdU double-labeled cells; closed arrowheads, PCNA-immunopositive cells with EdU/BrdU double-labeling; open arrows, PCNA-immunopositive cells with EdU single-labeling; closed arrows, PCNA-immunopositive cells with BrdU single-labeling.

Immunostaining for cCasp3, an apoptotic marker [32,33], was observed in EdU+, BrdU+, and EdU+/BrdU+ cells (Figure 4A; Table 1), suggesting that SVZ progenitors, including the bRG, experienced programmed cell death. There was no significant difference in the proportion of cCasp3 immunostaining in these cells between the LPS-exposed and control ferrets. Therefore, LPS exposure could not induce apoptosis in post-proliferative (EdU+) and/or proliferating (BrdU+) SVZ progenitors (Table 1).

#### 2.3.2. Proportion of PCNA Immunostaining

PCNA is a proliferation marker that is retained for a short time after cell cycle exit [33]. PCNA immunostaining was observed in SVZ progenitors in both the iSVZ and oSVZ (Figure 4B). A small population of EdU+ cells (6.1–14.5%) was PCNA-immunopositive in both the LPS-exposed and control ferrets (Table 1). The proportion of PCNA immunopositivity in BrdU+ cells was significantly lower in the iSVZ of LPS-exposed ferrets (27.2%) than in the controls (61.7%) (*p* < 0.05) (Table 1). Thus, PCNA expression remained in SVZ progenitors after proliferation. The reduced percentage of PCNA immunostaining in BrdU+ cells in LPS-exposed ferrets suggests that LPS attenuates PCNA expression in SVZ progenitors following proliferation, which is possibly directed toward neural differentiation.

#### 2.3.3. Proportion of Ki67 and PH3 Immunostaining

**Figure 5:**
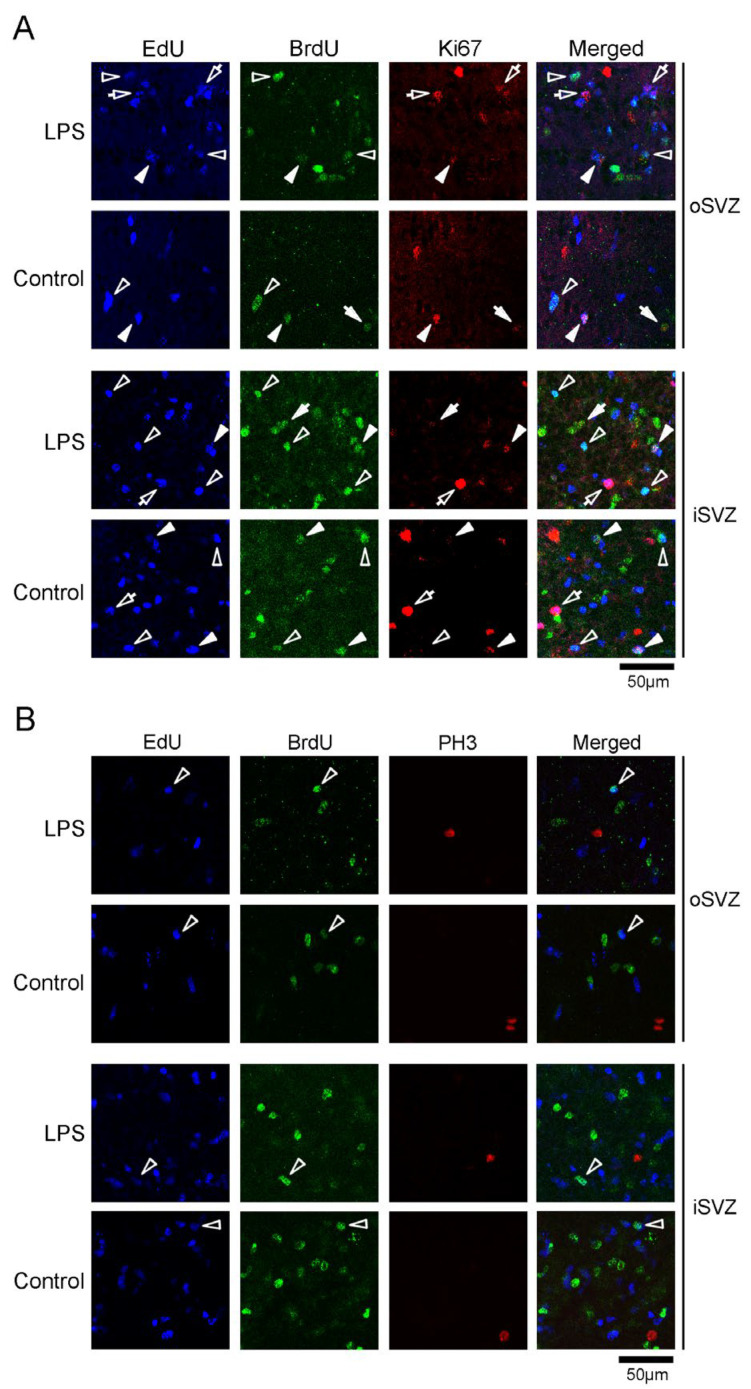
Immunofluorescence for cell cycle markers Ki67 and phosphohistone H3 (PH3) with EdU and BrdU labeling in the premature cortex of the LPS-exposed and control ferrets on postnatal day 7. Positions for capturing immunofluorescent images are shown in Figure S1B. (**A**) Ki67 immunofluorescence with EdU and BrdU labeling in the outer and inner subventricular zones (oSVZ and iSVZ). Open arrowheads, EdU/BrdU double-labeled cells; closed arrowheads, Ki67-immunopositive cells with EdU/BrdU double-labeling; open arrows, Ki67-immunopositive cells with EdU single-labeling; closed arrows, Ki67-immunopositive cells with BrdU single-labeling. (**B**) PH3 immunofluorescence with EdU and BrdU labeling in the oSVZ and iSVZ. Open arrowheads, EdU/BrdU double-labeled cells.

Immunostaining for cell cycle markers Ki67 and PH3 [34] was found in the SVZ progenitors of both the iSVZ and oSVZ (Figure 5A,B). Ki67 immunostaining was observed in EdU+, BrdU+, and EdU+/BrdU+ cells ranging from 2.8% to 17.2%, without significant differences in incidence between the LPS-exposed and control ferrets (Table 1). The percentage of Ki67 immunostaining was higher in EdU+/BrdU+ cells than in EdU+ and BrdU+ cells, ranging from 17.4% to 44.4% (Table 1). PH3 immunostaining was rarely observed (0.5–5.0%) in EdU+ cells and was absent in BrdU+ and EdU+/BrdU+ cells (Table 1). Thus, the expressions of cell cycle markers did not differ in the post-proliferative (EdU+) or proliferating (BrdU+) SVZ progenitors, including the self-renewed (EdU+/BrdU+) bRG, between LPS-exposed and control ferrets.

#### 2.3.4. Proportion of Immunostaining for Pax6, Tbr2, Olig2, Cux1, and Ctip2

**Figure 6:**
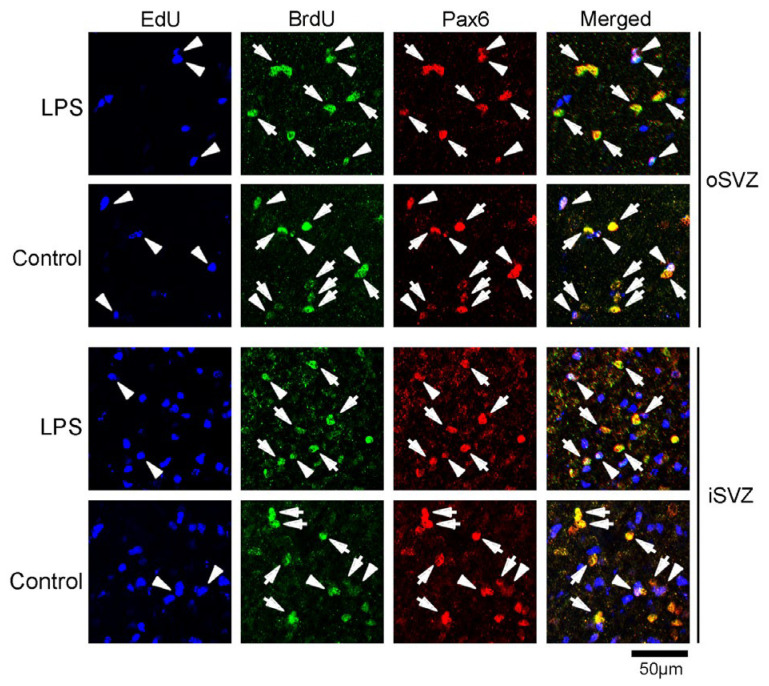
Immunofluorescence for Pax6 with EdU and BrdU labeling in the premature cortex of the LPS-exposed and control ferrets on postnatal day 7. Positions for capturing immunofluorescent images are shown in Figure S1B. Closed arrowheads, Pax6-immunopositive cells with EdU/BrdU double-labeling; closed arrows, Pax6-immunopositive cells with BrdU single-labeling. iSVZ, inner subventricular zone; oSVZ, outer subventricular zone.

Pax6 is a stable marker for bRG in mammals and is also expressed in IPs and apical radial glia [28,29]. These stem/progenitor cells can be distinguished by their location and/or the expression of other transcription factors, such as Tbr2 expression in IPs [28,29]. The proportion of Pax6 immunopositivity in EdU+ cells was 13.5% in the iSVZ and 20.0% in the oSVZ of LPS-exposed ferrets, which was significantly greater than that in the iSVZ (5.5%) and oSVZ (2.4%) of controls (Figure 6, Table 1). Although more than 85.0% of the BrdU+ and EdU+/BrdU+ cells were Pax6-immunopositive in both the iSVZ and oSVZ, a significantly greater proportion of Pax6 immunopositivity was found in the iSVZ of LPS-exposed ferrets (96.1%) compared with the controls (88.9%) (*p* < 0.05) (Figure 6, Table 1). Thus, LPS exposure increased Pax6 expression in either post-proliferative or proliferating SVZ progenitors.

#### 2.3.5. Proportion of Immunostaining for Tbr2, Olig2, Cux1, and Ctip2

**Figure 7:**
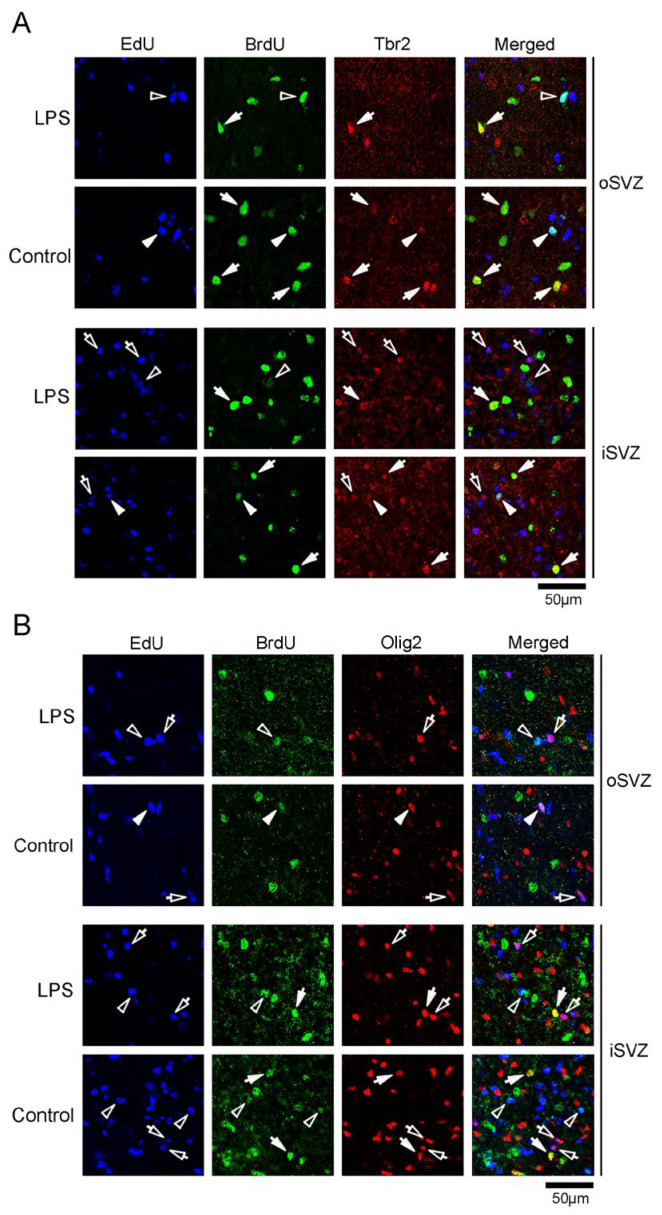
Immunofluorescence for Tbr2 and Olig2 with EdU and BrdU labeling in the premature cortex of the LPS-exposed and control ferrets on postnatal day 7. Positions for capturing immunofluorescent images are shown in Figure S1B. (**A**) Tbr2 immunofluorescence with EdU and BrdU labeling in the outer and inner subventricular zones (oSVZ and iSVZ). Open arrowheads, EdU/BrdU double-labeled cells; closed arrowheads, Tbr2-immunopositive cells with EdU/BrdU double-labeling; open arrows, Tbr2-immunopositive cells with EdU single-labeling; closed arrows, Tbr2-immunopositive cells with BrdU single-labeling. (**B**) Olig2 immunofluorescence with EdU and BrdU labeling in the oSVZ and iSVZ. Open arrowheads, EdU/BrdU double-labeled cells; closed arrowheads, Olig2-immunopositive cells with EdU/BrdU double-labeling; open arrows, Olig2-immunopositive cells with EdU single-labeling; closed arrows, Olig2-immunopositive cells with BrdU single-labeling.

**Figure 8:**
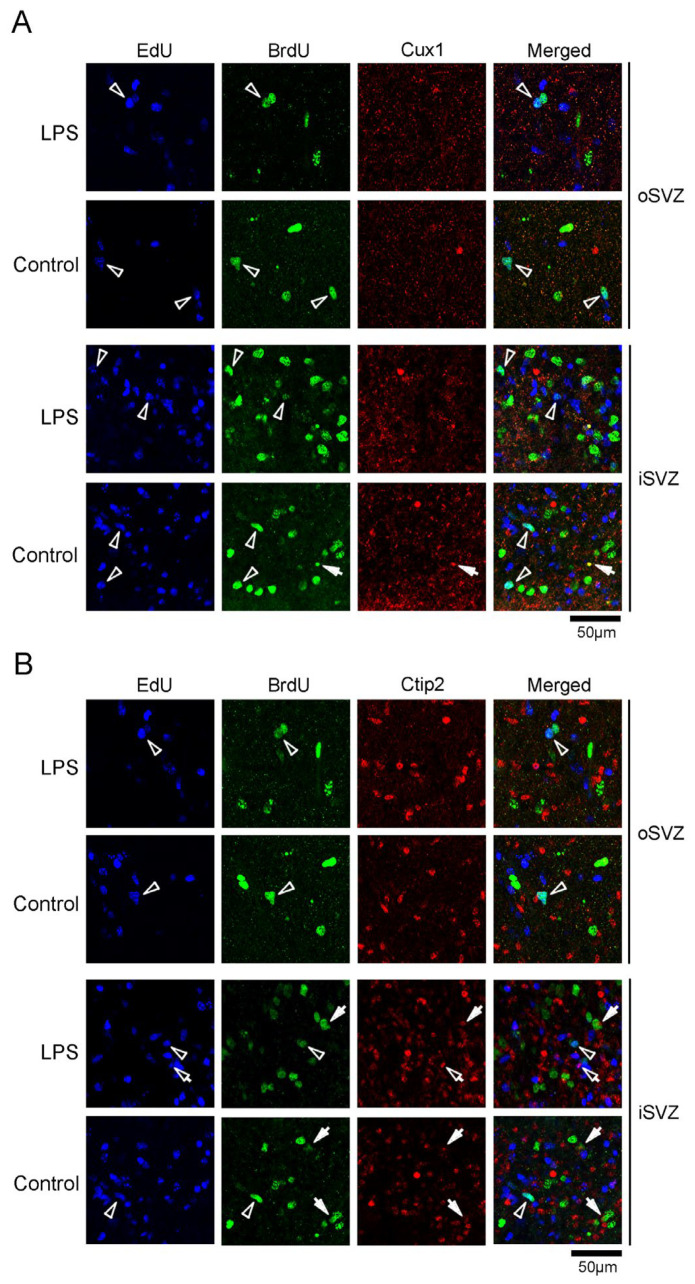
Immunofluorescence for Cux1 and Ctip2 with EdU and BrdU labeling in the premature cortex of the LPS-exposed and control ferrets on postnatal day 7. Positions for capturing immunofluorescent images are shown in Figure S1B. (**A**) Cux1 immunofluorescence with EdU and BrdU labeling in the outer and inner subventricular zones (oSVZ and iSVZ). Open arrowheads, EdU/BrdU double-labeled cells; closed arrows, Cux1-immunopositive cells with BrdU single-labeling. (**B**) Ctip2 immunofluorescence with EdU and BrdU labeling in the oSVZ and iSVZ. Open arrowheads, EdU/BrdU double-labeled cells; open arrows, Ctip2-immunopositive cells with EdU single-labeling; closed arrows, Ctip2-immunopositive cells with BrdU single-labeling.

EdU and BrdU labeling was also performed in the same series of sections immunostained for other marker antigens, such as Tbr2, a marker for IPs [28,29]; Olig2, which is expressed in progenitors with a glial progeny [29]; Cux1, a marker for postmitotic upper cortical layer neurons [35]; and Ctip2, a marker for postmitotic lower cortical layer neurons [36]. As the proportions of these marker antigens were independently estimated, the overlapping expression of each antigen was unclear. However, some BrdU+ and EdU+/BrdU+ cells were immunopositive for marker antigens, with the exception of Cux1 (Figure 7 and Figure 8), suggesting the co-expression of Pax6 with other antigens in proliferating SVZ progenitors, including the self-renewing bRG. Notably, significant differences in the proportions of Tbr2 and Ctip2 immunopositivity in BrdU+ cells were observed in the iSVZ between the LPS-exposed and control ferrets. BrdU+ cells exhibited a lower proportion of Tbr2 immunopositivity in LPS-exposed ferrets (22.2%) than in controls (42.6%) (*p* < 0.001), and a higher proportion of Ctip2 immunopositivity in LPS-exposed ferrets (22.2%) than in controls (8.6%) (*p* < 0.05) (Table 1). Thus, LPS exposure might facilitate the differentiation of Tbr2+ IPs into Ctip2+ immature neurons. Combining this finding with the BrdU+ cell density results (Figure 1B), LPS promoted the differentiative division of SVZ progenitors. Furthermore, LPS did not alter the proportion of immunostained neurogenesis markers in EdU+ cells, suggesting no influence of LPS on the differentiation of post-proliferative SVZ progenitors. In contrast, EdU+/BrdU+ cells in both the iSVZ and oSVZ were Pax6+ (>85%) and Tbr2+ (30.0–50.0%), without significant differences between the LPS-exposed and control ferrets (Table 1). EdU+/BrdU+ cells experienced two rounds of cell division at a 48 h interval, leading to labeling of the self-renewing bRG [31]. Therefore, some bRG might transform into Tbr2+ IPs immediately following their self-renewal, apart from the effects of LPS.

### 2.4. Densities of Cells Immunostained for Various Marker Antigens

**Figure 9:**
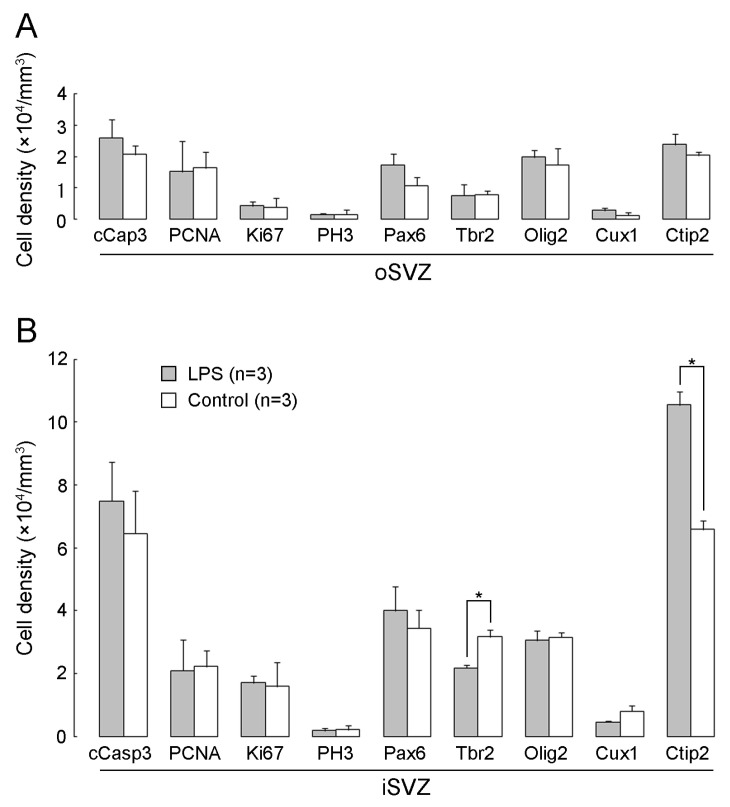
Bar graphs of densities of cells immunostained for various marker antigens in the premature cortex of the LPS-exposed and control ferrets on postnatal day 7. (**A**) Outer subventricular zone (oSVZ). (**B**) Inner subventricular zone (iSVZ). cCasp3, cleaved caspase 3; PH3, phosphohistone H3. Data are shown as means ± standard deviations. Significance is indicated using Scheffe’s test at * *p* < 0.001; number of ferrets = 3 per group.

Two hours after the second LPS exposure on PD 7, a significant difference in cell density between the LPS-exposed and control ferrets was observed in Tbr2+ and Ctip2+ cells in the iSVZ but not in the oSVZ (Figure 9A,B). Tbr2 immunostaining was observed in IPs in both the iSVZ and oSVZ [28,29]. In contrast, Pax6 is a stable marker for the bRG in mammals and is also expressed in IPs [28,29]. Thus, IPs can be defined as progenitor populations in the SVZ that are distinct from the bRG by Tbr2 immunostaining. The density of Tbr2+ IPs in the iSVZ was significantly lower in LPS-exposed ferrets than in controls as per the results of Scheffe’s test (*p* < 0.001), following significant effects of LPS exposure (*F_[1,4]_* = 12.774, *p* < 0.05), SVZ regions (*F_[1,4]_* = 141.710, *p* < 0.001), and an interaction between these two factors (*F_[1,4]_* = 10.837, *p* < 0.05) using the two-way repeated-measures ANOVA (Figure 9B). The most abundant cell populations, particularly in the iSVZ, were Ctip2+ immature neurons. Complementary to the lower density of Tbr2+ IPs, Ctip2+ immature neurons were significantly denser in the iSVZ of the LPS-exposed ferrets compared to controls according to the results of Scheffe’s test (*p* < 0.001), following significant effects of LPS exposure (*F_[1,4]_* = 78.995, *p* < 0.001), SVZ regions (*F_[1,4]_* = 1168.401, *p* < 0.001), and an interaction between these two factors (*F_[1,4]_* = 94.840, *p* < 0.001) using the two-way repeated-measures ANOVA (Figure 9B). There were no significant differences in the densities of cells immunostained for other marker antigens, such as Pax 6, Cux1, and Olig2, between the LPS-exposed and control ferrets. The changes in densities of Tbr2+ IPs and Ctip2+ immature neurons in the iSVZ after LPS exposure may indicate the LPS-promoted differentiative division of SVZ progenitors. This is supported by the increased density of BrdU+ cells (Figure 3B) and alterations in the proportions of Tbr2 and Ctip2 immunostaining (Table 1). cCasp3+ cells were distributed throughout the iSVZ to the oSVZ in both LPS-exposed and control ferrets without a significant difference in their densities (Figure 9A,B). Thus, some SVZ progenitors experienced naturally occurring programmed cell death under physiological conditions, whereas LPS exposure did not alter cCasp3+ cell density. There was no difference in the densities of cells expressing proliferation/cell cycle markers, such as PCNA, Ki67, and PH3, in the iSVZ and oSVZ between the LPS-exposed and control ferrets (Figure 9A,B).

## 3. Discussion

LPS is a natural agonist of TLR4, which is expressed in neurons and various types of glial cells, such as microglia, oligodendrocytes, and astrocytes, in the CNS [37] and plays a crucial role in inflammatory responses to CNS diseases and injury [38]. TLR4 is also expressed in neural stem/progenitor cells and modulates adult hippocampal neurogenesis [22,26] and neurogenesis during brain repair following injury [23,24,25,27]. Grasselli et al. (2018) [39] reported that LPS facilitates the proliferation and differentiation of human neural stem cells derived from the telencephalic area of the fetal brain through TLR4 activation *in vitro*. We also report that LPS facilitates the proliferation of SVZ progenitors, including IPs, and the differentiation of IPs into Ctip2+ immature neurons at a later stage of cortical neurogenesis. Such LPS-promoted differentiative division of SVZ progenitors may also be TLR4-related.

Our study revealed no alteration in apoptosis of SVZ progenitors in the developing ferret cortex immediately after LPS exposure. TLR4 inhibition by FP7, a synthetic TLR4 antagonist, induces apoptosis in human neural stem cells [39]. In contrast, LPS can induce neuronal apoptosis through astrocyte-mediated inflammatory responses [40] and can upregulate various factors, such as podoplanin [41], promyelocytic leukemia zinc finger [42], and PR domain-containing 5 [43], in cerebral cortical neurons. TLR4 expression is downregulated during neural stem cell differentiation [39], suggesting that LPS protects neural stem/progenitor cells from apoptosis by activating TLR4 signaling.

Prenatal exposure to LPS is widely used for producing MIA models in rodents, which show schizophrenia-like brain abnormalities [7,44,45]. In human patients with schizophrenia, abnormal cortical gyrification is associated with altered cortical thickness, lower gray matter volume, and disrupted neural connectivity [15,17]. While neurodevelopmental events, such as cortical neurogenesis from the bRG and the onset of sulcogyrogenesis, typically occur during mid-gestation in humans and non-human primates [28,29,46], ferrets undergo these events during the first weeks after birth [13,14,28,29]. Thus, administering LPS to ferrets at PDs 6 and 7, as performed in this study, may be appropriate for creating an MIA model when extrapolating these findings to humans.

Our previous study revealed that VPA exposure in ferret infants during late cortical neurogenesis caused the proliferation of SVZ progenitors, followed by their differentiation into Cux1+ immature neurons [21], resulting in altered sulcal infolding in multimodal-associated cortical regions by modifying the cortical thickness of the sulcal bottoms [20]. In the present study, we exposed ferret infants to LPS using the administration schedule practiced in the VPA-exposure experiments [20,21]. LPS exposure had a proliferative effect on Pax6+ SVZ progenitors in ferret infants, similar to that of VPA exposure, but it facilitated the differentiation of IPs into Ctip2+ immature neurons, which was unlike that for VPA exposure. Such induction of different types of cortical neurons between these two agents might result in the migration of cells to distinct cortical layers, such as Cux1+ immature neurons migrating to the upper cortical layers [35] and Ctip2+ immature neurons migrating to the lower cortical layers [36]; moreover, this was expected, considering that LPS exposure causes gyrification abnormalities that are distinct from those caused by VPA exposure. Additionally, prenatal exposure to LPS causes histoarchitectural alterations in the mature cerebral cortex, such as overall hypomyelination [8,10], reduced parvalbumin+ neurons in the prefrontal cortex [8,11], and abnormal organization of minicolumns in the anterior cingulate and somatosensory cortices [9] of mouse and rat offspring. These corticoarchitectural changes are small but associated with impairments in social behavior and cognition [8,9,10,11]. Future research is needed to characterize the gyrification abnormalities and their related histoarchitectural changes in the mature cortex of our LPS-exposed ferret model.

This study did not evaluate microglial activation in the iSVZ and oSVZ of the premature cortex of ferret neonates immediately after LPS exposure. Systemic exposure to LPS increased microglial activation, characterized by mixed proinflammatory (M1) and anti-inflammatory (M2) phenotypes [47], with the production of proinflammatory cytokines, such as TNF-α, interleukin 1β, and IL-6 in rat neonates [47,48]. Although proinflammatory cytokines released from activated microglia are involved in neurogenesis and oligodendrogenesis [49], their effects on neural stem/progenitor cells vary depending on their concentration [48]. It is difficult to assess LPS-mediated production of proinflammatory cytokines in microglia using in vivo experiments, such as in the current study. This is a limitation in the current study. In vitro experiments, such as neurosphere assays, are needed to investigate the involvement of microglial activation in the LPS-related neurogenesis of SVZ progenitors.

## 4. Experimental Procedures

### 4.1. Animals

Five pregnant ferrets were purchased from Japan SLC (Hamamatsu, Japan), and their six naturally delivered pups were used. The pups were reared with lactating mothers (3–5 pups/mother) in stainless-steel cages (80 cm × 50 cm × 35 cm) maintained at 21.5 ± 2.5 °C under 12 h artificial illumination in the Facility of Animal Breeding, Nakaizu Laboratory, Japan SLC. All lactating mothers were fed a pellet diet (High-Density Ferret Diet 5L14; PMI Feeds, Inc., St. Louis, MO, USA) and tap water *ad libitum*.

The schedule for the thymidine analog and LPS administration was designed as described in a previous study [31]. Six male ferret pups were intraperitoneally injected with EdU at 30 µg/g body weight (Sigma-Aldrich, St. Louis, MO, USA) on PD 5 and BrdU at 30 µg/g body weight (Sigma-Aldrich) on PD 7. Three pups were intraperitoneally administered LPS at 500 µg/g body weight on PDs 6 and 7. The second LPS injection was done at the same time as the BrdU injection. This administration schedule covered two rounds of cell division at a 48 h interval, leading to self-renewing bRG labeling [31]. The remaining three pups that were not given LPS exposure were used as controls. Two hours after the BrdU injection, all animals were perfused with 4% paraformaldehyde (PFA) in PBS under deep anesthesia with ~2% isoflurane gas. The mean body weight of PD 7 ferrets was 20.4 ± 2.2 g in the LPS-exposed group (*n* = 3), which was significantly lower than the 32.0 ± 3.0 g body weight in the age-matched controls (*n* = 3) (*p* < 0.05, Student’s *t*-test).

### 4.2. Immunohistochemical Procedures

Cerebral hemispheres were immersed in a 30% sucrose–PBS solution overnight and then embedded in an optimal cutting temperature compound (Sakura Finetek Japan Co., Ltd., Tokyo, Japan) at −70 °C. Coronal cryosections of the hemispheres were made at 100 µm thickness using a Retratome (REM-700; Yamato Koki Industrial Co. Ltd. Asaka, Japan) equipped with a refrigeration unit (Electro Freeze MC-802A; Yamato Koki Industrial, Asaka, Japan). All sections were collected in vials containing 4% PFA solution.

Serial coronal sections taken around the level of the anterior commissure were subjected to immunofluorescence and EdU detection. These sections included large expansions of both the iSVZ and oSVZ, and the examined cortical region corresponded to the primary somatosensory cortex [21]. The sections that were not used for immunohistochemical analysis were stained using hematoxylin for gross histological evaluation. All procedures were performed on floating sections. Sections were heated in the Antigen Unmasking Solution H-3300, pH 6.0 (Vector Labs Inc., Burlingame, CA, USA) for 30 min in a 90 °C water bath and then cooled at 4 °C for 30 min. Two hours following preincubation with PBS containing 0.1% Triton-X 100 (Sigma-Aldrich) at 37 °C for 1 h, immunofluorescence staining with EdU detection was carried out using the Click-iT reaction cocktail (Click-iT EdU Alexa Fluor 488 Imaging Kit, Thermo Fisher Scientific, Waltham, MA, USA) as reported previously [31]. The primary and secondary antibodies used in this study are listed in Tables S1 and S2. The primary antibodies used were highly specific for ferret brain tissue [21,28,31,50].

### 4.3. Estimation of Cell Density

Serial digital sectioning images were acquired at a 10 μm depth (section plane thickness, 1 μm; number of sections, 10) from the most superficial plane, the locations where EdU and BrdU labeling with immunostaining for various markers were obtained. Images were captured using an Axio Imager M2 ApoTome.2 microscope with a 20× objective, equipped with an AxioCam MRm camera (Zeiss, Gottingen, Germany) with the Zen 2.3 blue edition software (Zeiss). A set of sectional images 4 μm apart in the Z-direction (third and seventh from the superficial slices of the acquired images) were selected as the lookup and reference images, respectively. The dissector method using systematic random sampling was used to estimate the density of the immunostained and EdU- and/or BrdU-labeled cells, as reported previously [31]. In the sections immunostained for each marker, frames with 24 square boxes (box size 40 μm × 40 μm) were used from one section (the left and right hemispheres) to systematically select the region of interest (ROI) randomly superimposed on the iSVZ and oSVZ of both the lookup and reference images, at the same positions perpendicular to the ventricular surface. Thymidine analog-labeled or immunostained cells were counted within the ROIs using the “forbidden line” rule. Their densities were calculated using the following formula: [Cell density = Qn−/(a × b × t)] (Qn− = total number of thymidine analog-labeled and/or immunostained cells appearing within ROIs in the lookup images, but not in the reference images; a = 24, total number of ROIs in the lookup images from two sections (the left and right hemispheres) per animal; b = 40 μm × 40 μm area of counting box; and t = 4 μm, distance between the lookup and reference images). The proportions of immunostaining for various makers in EdU- and/or BrdU-labeled cells were estimated by summing the number of cells counted within all ROIs from all animals in each group. As the proportion was estimated independently for each marker antigen, the overlapping expression of each antigen was unclear.

### 4.4. Statistical Analysis

Measurements from the left and right hemispheres were combined, and the number of animals (*n*) was set to “3” in each group. Significant differences in the weights of the brains of LPS-exposed and control ferrets were statistically assessed using a one-way ANOVA followed by a two-tailed Student’s *t*-test. The densities of the immunostained and EdU- and/or BrdU-labeled cells were statistically analyzed using the two-way repeated-measures ANOVA, with “region” (iSVZ and oSVZ) and “group” (LPS-exposed and control groups) as factors. For post hoc testing, Scheffe’s test was used to detect significant differences in group and/or region × group interactions using the two-way repeated-measures ANOVA and simple main effects. The percentage of cells immunolabeled for markers among the thymidine analog-labeled cells was statistically assessed using the chi-square test. The total number of EdU single-, BrdU single-, and EdU/BrdU double-labeled cells was defined as “*n*” for the chi-square test.

## 5. Conclusions

Ferrets exhibit cerebral cortical development similar to that in humans and non-human primates, rather than rodents, in terms of the abundant emergence of the bRG in the SVZ and a massive expansion of the cortex with gyrification [13,14,28,29,30]. The main findings of the current investigation are that LPS facilitated the proliferation of SVZ progenitors, followed by the differentiation of Tbr2+ IPs into Ctip2+ immature neurons. Such LPS-promoted differentiative division of SVZ progenitors may be involved in the activation of TLR4 signaling rather than in microglial activation and the production of proinflammatory cytokines. Our findings will help to elucidate the structural and functional abnormalities of the brain, such as altered cortical folding/infolding and social behavioral deficits, caused by innate immune activation by bacterial infections during cerebral cortical neurogenesis.

## Figures and Tables

**Table 1:** Percentages of immunostained cells for various markers in EdU single-, BrdU single- and EdU/BrdU double-labeled cells in subventricular zone of premature cortex.

	iSVZ		oSVZ	
	LPS	Control	LPS	Control
EdU single-labeled cells				
% of cleaved caspase 3+	13.7% (18/131)	9.3% (15/161)	32.6% (15/46)	50.0% (9/18)
% of PCNA+	6.1% (8/132)	8.8% (14/160)	14.5% (8/55)	9.5% (4/42)
% of Ki67+	17.2% (25/145)	12.6% (23/182)	6.5% (3/46)	10.0% (4/40)
% of PH3+	0.7% (1/145)	0.5% (1/182)	0% (0/46)	5.0% (2/40)
% of Pax6+	13.5% (19/141) *	5.5% (7/127)	20.0% (7/35) *	2.4% (1/42)
% of Tbr2+	10.6% (14/132)	13.8% (22/160)	3.6% (2/55)	9.5% (4/42)
% of Olig2+	10.6% (15/141)	7.9% (10/127)	17.1% (6/35)	23.8% (10/42)
% of Cux1+	0.7% (1/140)	4.2% (6/144)	2.0% (1/49)	0% (0/27)
% of Ctip2+	27.9% (39/140)	23.6% (34/144)	8.2% (4/49)	11.1% (3/27)
BrdU single-labeled cells				
% of cleaved caspase 3+	10.9% (10/92)	21.2% (11/52)	32.4% (11/34)	13.6% (3/22)
% of PCNA+	27.2% (22/81) *	61.7% (29/47)	62.5% (20/32)	71.4% (20/28)
% of Ki67+	16.9% (11/65)	10.9% (5/46)	2.8% (1/36)	8.3% (1/12)
% of PH3+	0% (0/65)	0% (0/46)	0% (0/36)	0% (0/12)
% of Pax6+	96.1% (124/129) *	88.9% (80/90)	98.1% (51/52)	100% (25/25)
% of Tbr2+	22.2% (18/81) **	42.6% (20/47)	40.6% (13/32)	39.2% (11/28)
% of Olig2+	8.5% (11/129)	7.8% (7/90)	17.3% (9/52)	20.0% (5/25)
% of Cux1+	4.9% (4/81)	5.2% (3/58)	9.1% (3/33)	0% (0/21)
% of Ctip2+	22.2% (18/82) *	8.6% (5/58)	6.1% (2/33)	4.8% (1/21)
EdU/BrdU double-labeled cells				
% of cleaved caspase 3+	13.3% (2/15)	11.1% (1/9)	66.7% (4/6)	50.0% (4/8)
% of PCNA+	55.6% (5/9)	80.0% (12/15)	70.0% (7/10)	91.7% (11/12)
% of Ki67+	25.0% (9/36)	31.6% (6/19)	17.4% (4/23)	44.4% (4/9)
% of PH3+	0% (0/36)	0% (0/19)	0% (0/23)	0% (0/9)
% of Pax6+	100% (18/18)	100% (41/41)	85.0% (17/20)	100% (16/16)
% of Tbr2+	33.3% (3/9)	46.7% (7/15)	30.0% (3/10)	50.0% (6/12)
% of Olig2+	22.2% (4/18)	9.8% (4/41)	30.0% (6/20)	37.5% (6/16)
% of Cux1+	0% (0/10)	0% (0/6)	0% (0/2)	0% (0/4)
% of Ctip2+	20.0% (2/10)	16.7% (1/6)	0% (0/2)	0% (0/4)

Percentages are calculated by summing each labeled cell counted within all ROIs from cerebral cortex of three ferrets. The number of each labeled cell for calculating the percentages is shown in parentheses. * *p* < 0.05, ** *p* < 0.001 vs. controls (chi-square test).

## Data Availability

The original contributions presented in this study are included in the article/Supplementary Materials. Further inquiries can be directed to the corresponding author.

## Supplementary Materials

The following supporting information can be downloaded at: https://www.mdpi.com/article/10.3390/ijms241914962/s1.
