# Supplementary material for: Neonatal Exposure to Lipopolysaccharide Promotes Neurogenesis of Subventricular Zone Progenitors in the Developing Neocortex of Ferrets
Source: Int J Mol Sci. 2023 Oct 6;24(19):14962. doi: 10.3390/ijms241914962 (PMC10573966; doi:10.3390/ijms241914962)
Supplement: Supplementary file 1 [file ijms-24-14962-s001.zip › Figure_S1.pdf]

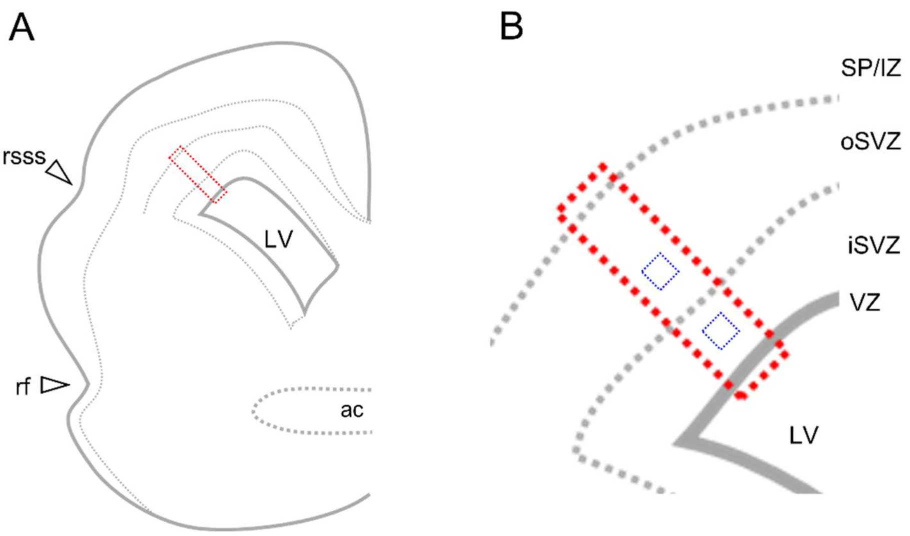

Figure S1. Positions for capturing immunofluorescent images. (A) An Illustration of coronal image at the level of the anterior commissure (ac). Red doted rectangle indicates the position for capturing immunofluorescent images shown in Figure 2 that include the inner subventricular zone (iSVZ) through the outer subventricular zone (oSVZ). (B) An enlarged image around the iSVZ and oSVZ in the illustration A. Blue rectangles indicate the positions for capturing high-magnification immunofluorescent images of the iSVZ and oSVZ shown in Figures 2, 4-8. IZ, intermediate zone; LV, lateral ventricle; rf, rhinal fissure; rsss, rostral suprasylvian sulcus; SP, subplate; VZ, ventricular zone.
