# Supplementary material for: Neonatal Exposure to Lipopolysaccharide Promotes Neurogenesis of Subventricular Zone Progenitors in the Developing Neocortex of Ferrets
Source: Int J Mol Sci. 2023 Oct 6;24(19):14962. doi: 10.3390/ijms241914962 (PMC10573966; doi:10.3390/ijms241914962)
Supplement: Supplementary file 1 [file ijms-24-14962-s001.zip › Table_S1_R.pdf]

Table S1. Primary antibodies used in the study.

Antigens	Sources	Species	Cat#	RRID	Concentration used	References
BrdU	Abcam	Sheep polyclonal	ab1893	AB_302659	1:500	Sawada (2022) Int. J. Mol. Sci.
Cleaved caspase 3	GeneTex Inc.	Rabbit polyclonal	GTX22302	AB_384753	1:500	Sawada(2019) IBRO Rep.
PCNA	Merck Millipore	Mouse monoclonal	MAB424	AB_95106	1:500	Kelava et al. (2012) Cereb. Cortex<br>Kamiya & Sawada (2021) Anat. Rec.
Ki67	Dako	Mouse monoclonal	M7240	AB_2142367	1:500	Turrero Garcia et al. (2015) J. Comp. Neurol.
PH3	Merck Millipore	Rabbit polyclonal	06-570	AB_310177	1:500	Kelava et al. (2012) Cereb. Cortex<br>Martínez-Cerdeño et al. (2012) PLoS One
Pax6	Abcam	Mouse monoclonal	ab78545	AB_1566562	1:500	Martínez-Cerdeño et al. (2012) PLoS One<br>Sawada (2019) IBRO Rep.
Tbr2	Abcam	Rabbit polyclonal	ab23345	AB_778267	1:200	Kelava et al. (2012) Cereb. Cortex
Olig2	IBL	Rabbit polyclonal	18953	AB_1630817	1:500	Sawada (2019) IBRO Rep.
Cux1	Santa Cruz Biotech. Inc	Mouse monoclonal	SC-514008	AB_2715519	1:200	Sawada (2022) Int. J. Mol. Sci.
Ctip2	Abcam	Rat monoclonal	ab18465	AB_2064130	1:200	Sawada (2022) Int. J. Mol. Sci.
