# Supplementary material for: Neonatal Exposure to Lipopolysaccharide Promotes Neurogenesis of Subventricular Zone Progenitors in the Developing Neocortex of Ferrets
Source: Int J Mol Sci. 2023 Oct 6;24(19):14962. doi: 10.3390/ijms241914962 (PMC10573966; doi:10.3390/ijms241914962)
Supplement: Supplementary file 1 [file ijms-24-14962-s001.zip › Table_S2.pdf]

Table S2. Secondary antibodies used in the study.

Secondary antibodies	Source	Cat#	Concentration used
Alexa 555-labeled donkey anti-rabbit IgG	Thermo Fisher	A-31572	1:500
Alexa 555-labeled donkey anti-mouse IgG	Thermo Fisher	A-31570	1:500
Alexa 647-labeled chicken anti-rat IgG	Thermo Fisher	A- 21472	1:500
Alexa 647-labeled donkey anti-sheep IgG	Abcam	ab150179	1:500
